# Feline Toxoplasmosis in Greece: A Countrywide Seroprevalence Study and Associated Risk Factors

**DOI:** 10.3390/pathogens11121511

**Published:** 2022-12-09

**Authors:** Georgios Sioutas, Isaia Symeonidou, Athanasios I. Gelasakis, Christos Tzirinis, Elias Papadopoulos

**Affiliations:** 1 Laboratory of Parasitology and Parasitic Diseases, School of Veterinary Medicine, Faculty of Health Sciences, Aristotle University of Thessaloniki, 54124 Thessaloniki, Greece; 2 Laboratory of Anatomy and Physiology of Farm Animals, Department of Animal Science, School of Animal Biosciences, Agricultural University of Athens, 11855 Athens, Greece

**Keywords:** *Toxoplasma gondii*, antibodies, cats, seroprevalence, Greece, risk factors, One Health, IgG, immunochromatographic test

## Abstract

*Toxoplasma gondii* is a ubiquitous zoonotic parasite, with felines being the only definitive hosts. Cats shed oocysts with their faeces, and seroprevalence studies can be used to indirectly assess the environmental contamination. The current study aimed to evaluate *T. gondii* seroprevalence in Greek cats and identify possible risk factors. In total, 1554 blood samples were analyzed from different cats across all nine geographic regions of Greece, and a short questionnaire was completed for each cat. A rapid immunochromatographic test was used to detect anti-*T. gondii* antibodies, IgG type, and 21.8% of cats were seropositive. Regarding risk factors, when chi-square tests were applied, seropositivity was significantly higher (*p* < 0.05) in rural cats, cats with outdoor access, and hunting cats. Gender, age, ownership, and raw feeding were not significant risk factors, although female, adult, stray, and raw-feeding cats had a higher seroprevalence than their counterparts. Binary logistic regression models were developed to adjust for the confounding effects of the initially recognized risk factors, and only hunting in urban areas remained a significant risk factor. Greek cats had lower seropositivity than the average European value, and the present research highlights the importance of updated seroprevalence and risk factor studies within the context of One-Health.

## 1. Introduction

*Toxoplasma gondii* (Phylum: Apicomplexa, Family: Sarcocystidae) is a ubiquitous, protozoan, coccidian, obligate intracellular parasite [1]. It has a facultative indirect life cycle with felids (i.e., domestic cats) as definitive hosts [2]. This protozoon can infect almost all homeothermic animals, including humans, who serve as intermediate hosts [3]. Regarding its life cycle, there are three infective stages: sporozoites, tachyzoites, and bradyzoites [4]. The most common infection routes are the ingestion of sporulated oocysts via water, soil, and raw or unwashed fruits and vegetables contaminated with infected cat faeces [5]; the ingestion of tissue cysts in raw or undercooked meat of infected animals; and the transplacental transmission of tachyzoites [6]. Cats typically become infected early in their life from ingesting cysts while hunting [7] and shed-resistant oocysts with their faeces [7]. Overall, toxoplasmosis in cats has a low morbidity and mortality rate [8], and the majority of cats are asymptomatic [7]. Intermediate hosts display symptoms such as cerebral disease [9] in immunosuppressed patients [10] and abortions [11,12,13]. Approximately one-third of the world’s human population is chronically infected with *T*. *gondii* [14]. This protozoon is ranked number three out of all food-borne hazards in Europe, in terms of disease burden, according to the World Health Organization [15], and congenital toxoplasmosis is the second most common congenital disease in humans [16]. Felidae, as the only definitive host, play a highly significant role in the epidemiology of toxoplasmosis [8].

Conclusive diagnosis in cats is rather difficult [17]. The serological identification of *T. gondii*-specific humoral antibodies is a widely used and reliable method [18,19] and is recommended in epidemiological studies [20]. Immunochromatographic tests (ICTs) represent an easy, practical, low-cost, and rapid screening method commonly used in field population studies without sophisticated equipment [21,22]. They can be employed to detect IgG antibodies in cats, and their high specificity and sensitivity compared to ELISA and LAT render them a reliable option [21,22,23]. Seropositive cats have already excreted oocysts with their faeces [24], while assessing the contamination of the environment with oocysts is impractical for technical reasons [25,26,27]. Hence, seroprevalence studies in domestic cats are very important for public health practitioners and veterinarians. They can be used to indirectly evaluate the environmental oocyst burden and consequently estimate the infection pressure to both people and animals. Until now, Greece remained one of the few countries in the E.E. without any relevant seroprevalence data for cats; therefore, the actual feline prevalence of *T. gondii* was vastly underrated [28,29]. Towards this end, the objectives of this novel study were to estimate the seroprevalence and assess associated risk factors of toxoplasmosis in cats in Greece.

## 2. Materials and Methods

### 2.1. Collection of Blood Samples and Study Area

All 1554 cat (*Felis silvestris catus*) blood samples were collected and tested during the summer of 2022 at the Laboratory of Parasitology and Parasitic Diseases, School of Veterinary Medicine, Faculty of Health Sciences, Aristotle University of Thessaloniki, Greece. The study area included all nine geographical regions of Greece (Thrace, Macedonia, Epirus, Thessaly, Central Greece, Peloponnese, Crete, Ionian islands, and Aegean islands). Cats included in this study were selected randomly and were clinically healthy. Owners or people from various animal welfare organizations brought the cats to private veterinary clinics around Greece for routine check-up examinations, vaccinations, or sterilizations, which required blood sampling. From each cat, 1 mL of blood was drawn by puncturing one jugular, cephalic, or femoral vein, and then transferred into EDTA-containing vials for later antibody testing. Each whole blood sample was stored in the refrigerator (+4 °C) until testing. A questionnaire was completed for each cat in order to collect relevant information regarding potential risk factors. In detail, the following were recorded:

Environment: Rural area (village) or urban area (city).

Gender: Female or male.

Age: Cat age was determined based on questionnaire answers, teeth development, and dental attrition (incisors, canines, premolars, and molars) [30]. Based on these data, cats were categorized into two groups reflecting age (those older than six months (>6 months) and those equal to or younger than six months (≤6 months)).

Raw feeding: Cats were categorized into two groups, those that additionally fed on a raw diet and those that fed strictly on commercial food.

Lifestyle: Cats were separated into those with outdoor access and those living strictly indoors.

Ownership: Cats were categorized into client-owned or stray-feral.

Hunting activity: Cats were split into two categories (those that hunt and those with no hunting activity).

This study was conducted in accordance with bioethics and animal welfare standards, and it was authorized by the Ethics Committee of the ARISTOTLE UNIVERSITY OF THESSALONIKI (192746/2022).

### 2.2. Immunochromatographic Test Kit

All blood samples were transported inside coolers with ice packs and examined at the Laboratory of Parasitology and Parasitic Diseases, Faculty of Health Sciences, School of Veterinary Medicine, Aristotle University of Thessaloniki, Greece. The ICT used (Anigen Rapid Toxoplasma Ab Test Kit, BioNote Inc., Hwaseong-si, Republic of Korea) is a reliable diagnostic kit (Sensitivity 100%, Specificity 99%) used for qualitatively detecting IgG antibodies against *T. gondii* in cats’ serum, plasma, or whole blood. Highly selective recombinant *T. gondii* antigens (GRA1 and SAG1) are used in the ICT, and the test’s principle is based on sandwich lateral flow immunochromatographic assay [31]. The steps outlined in the manufacturer’s instructions were precisely followed. Briefly, all reagents and samples were brought to room temperature before testing. The kit’s capillary tube was utilized, and 10 μL from each sample was added into the sample hole. Consequently, 3 drops of the assay diluents were added into the sample hole vertically. Results were interpreted at 10 min and no later than 20 min. If only one coloured line appeared in the control line region (C) and no line appeared in the test line region (T), then the test result was deemed as negative. If two lines appeared, one coloured line in the control line region (C), and another coloured line in the test line region (T), the test result was deemed positive. Faint lines in the regions mentioned above, (C) and (T), also implied a positive result. If no coloured line appeared in the control line region (C), for any reason, the test result was deemed invalid, and the sample was retested. A positive test result meant that the corresponding cat had been previously exposed to *T. gondii*. All result interpretations were based on the official brochure included with each test kit by the manufacturing company, and all whole blood samples were tested by the same person who was blind to each sample’s origin to avoid bias.

### 2.3. Statistical Analyses

Chi-square tests were performed, and odds ratios and relative risks were calculated to assess potential risk factors and the initial quantification of their association with seropositivity against *T. gondii*.

The seroprevalence of *T. gondii* infections was calculated, and its 95% confidence intervals (CI 95%) were estimated using the Wilson score interval method. A binary logistic regression model was used to assess the relationship between potential risk factors and the likelihood that a cat is seropositive to *T. gondii* in urban and rural areas separately. The aforementioned risk factors included age, sex, raw feeding, and hunting activity, as described in Model 1:

Y_G_ = α + β_1_Χ_1_ + β_2_Χ_2_ + β_3_Χ_3_ + β_4_Χ_4_ (Model 1)

where Y_G_ = the probability of a cat being seropositive for *T. gondii*, β_1_ to β_4_ the regression coefficients of age (X_1_, 0 = ≤6 months, 1 = >6 months), sex (X_2_, 0 = male, 1 = female), raw feeding (X_3_, 0 = no raw feeding, 1 = raw feeding), and hunting activity (X_4_, 0 = no hunting activity, 1= hunting activity).

The statistical significance of individual predictors was tested using the Wald χ^2^ statistic of their regression coefficients (βs). Goodness-of-fit for each individual model was assessed using the Hosmer–Lemeshow (H-L) test, as well as Cox and Snell R^2^ and Nagelkerke R^2^ indices. Statistical significance was set at the 0.05 level.

## 3. Results

### 3.1. Descriptive Statistical Analysis

In total, 1554 cat serum samples were examined from different areas of Greece, and 339 (21.8%) were seropositive. Seropositivity percentages for the different risk factor groups of the examined cats are summarized in Table 1.

### 3.2. In-Depth Risk Analyses

Overall, 21.8% (339/1554, CI 95% 19.8 to 23.9%) of the sampled cats were seropositive to *T. gondii*. Table 2 below summarizes the results of the chi-square tests as well as the odds ratios and relative risks.

The association between the studied risk factors forced into the model (sex, age, raw feeding, and hunting activity) and the seropositivity to *T. gondii* are presented below in Table 3.

The likelihood of *T. gondii* infection was lower (*B* = −0.82, *df* = 1, *p* < 0.01) for non-hunting cats in comparison to those who hunted in urban areas (*ca.* 2.3 times, CI 95%, 1.2 to 4.2). Similarly, a 1.3 times higher (CI 95%, 1 to 1.8 times) probability for *T. gondii* seropositivity was recorded for female cats in rural areas; however, this value was not statistically significant (*B* = −0.28, *df* = 1, *p* = 0.061).

### 3.3. Regional Seroprevalence

**Figure 1:**
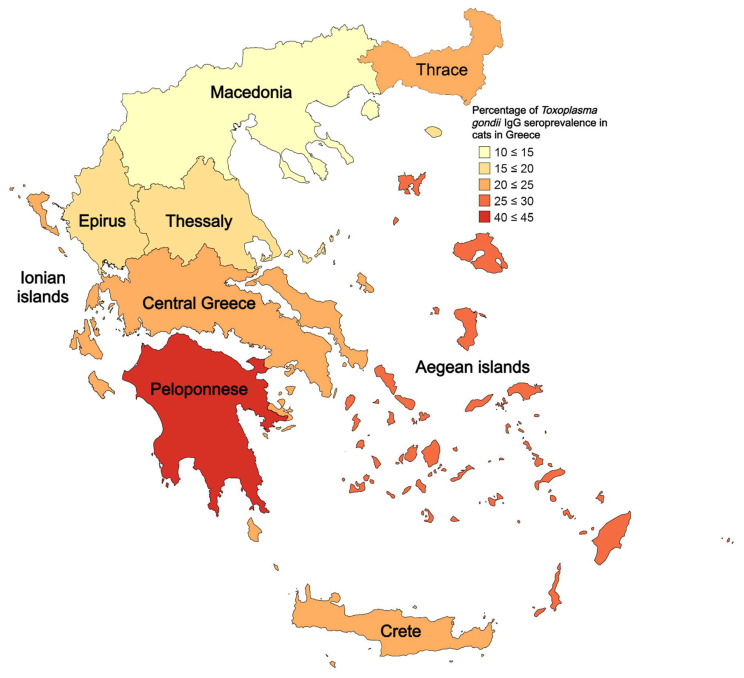
*Toxoplasma gondii* IgG seroprevalence rate in cats from different geographical regions of Greece.

The seroprevalence rate of cats in each geographical region is illustrated in Figure 1, and the exact percentages are given in Table 4.

## 4. Discussion

### 4.1. Diagnosis of T. gondii and Seropositivity Interpretation

Many diagnostic methods have been employed for the diagnosis of *T. gondii* infections in cats, each having benefits and drawbacks. Given that no diagnostic test has a sensitivity and specificity of 100%, this research provides novel insight into the seroprevalence of cats in Greece. Up to now, two recent countrywide studies in Greece reported 0 [28] to 0.4% [32] prevalence of *T. gondii*-like oocysts in the faeces of examined cats. In these studies, the identification of oocysts was achieved with a flotation technique that has two major drawbacks. Microscopic analysis requires the cat to be shedding oocysts when the faeces are collected, which is typically only seven days during its lifetime [33]. Research has demonstrated that only 0.4% of client-owned cats and 4.1% of feral cats pass oocysts at any given time in their faeces [34]. Additionally, *T. gondii* oocysts cannot be differentiated based on morphology of other feline coccidian oocysts such as *Hammondia* spp. [35] and *Besnoitia* spp. [36]. Therefore, in epidemiological studies such as the current one, the serological investigation of anti-*T. gondii* antibodies is more useful than evaluating the prevalence of *T. gondii*-like oocysts in the faeces of examined cats [33].

The total seroprevalence of *T. gondii* detected in this present study (21.8%) is lower than the worldwide pooled seropositivity of 37.5% [37] and the average seroprevalence in Europe which is even higher at 45.3% [37]. *T. gondii* seroprevalence ranges significantly across European countries, from 17.3% in Spain (client-owned and stray cats) [38] to 80.5% in Romania (client-owned and stray cats) [39]. In countries neighbouring Greece, such as Albania and Turkey, feline seropositivity reaches up to 42% (client-owned cats) [40] and 34.2% (stray cats) [41], respectively. Seroprevalence among the tested regions ranged from 14.9% in Macedonia to 42.8% in Peloponnese. It should be highlighted that the seropositivity of cats in different countries/regions is affected by a variety of factors such as cat ownership (client-owned or stray), sampling methods (inclusion/exclusion criteria), sampling size, cat age, diet, population density, environmental oocyst burden, test employed (different specificity and sensitivity), cut-off titres, and seropositivity in intermediate hosts that serve as prey (such as rodents) [42,43,44,45]. Therefore, caution should be exercised when comparing seroprevalence results from different countries. A wiser approach would be to track the seropositivity changes in one nation throughout the years using the same methodology. Research indicates that seroprevalence in a country is expected to drop through the years [45].

The Anigen Rapid Toxoplasma Ab Test Kit detects IgG antibodies which are produced in all infected cats and can be detected 4–6 weeks post-primary infection. IgG antibody titres peak 2–3 weeks after their first detection [46] and can persist for more than six years [47]. In general, cats usually seroconvert between the second and third week after ingesting bradyzoites, and by that time, they have already finished shedding oocysts. It is safe to assume that most seropositive cats no longer shed oocysts [33,47], and most likely will never shed oocysts again, except in instances, i.e., immunosuppression [48,49]. A positive IgG titre reflects a developed humoral immune response and indicates latent (chronic) toxoplasmosis [50]. On the flip side, seronegative cats may be sampled during the first two weeks of infection, and thus are excreting oocysts at that time, or may begin to excrete oocysts if they become infected [46]. Nonetheless, most IgG seropositive cats are asymptomatic [7,47]. In an attempt to interpret the recorded seroprevalence rate, high seropositivity in cats indicates that cats play a vital role in the *T. gondii* epidemiology in the specific country, as they already have spread a lot of oocysts [44]. On the other hand, low seropositivity, as in the case of this research, implies that there are many naive cats that might become infected and expel oocysts. Hence, these seronegative cats represent a greater future risk of contaminating the environment and transmitting the parasite to humans.

### 4.2. Risk Factors Break Down

Factors influencing *T. gondii* seropositivity in cats have been extensively studied but are not yet completely clarified, and further research is required [51]. Nonetheless, the current study provides insight into the various risk factors that can influence infection in cats living in Greece. Hunting was identified as the most significant risk factor for *T. gondii* infection using chi-square tests (*p* < 0.001) and remained the only significant risk factor in the binary logistic regression model for cats living in urban areas (*p* < 0.01). Many other studies have also identified hunting, i.e., rodents, who are the natural intermediate host, other small mammals, birds, and mechanical vectors, as a crucial risk factor for the consumption of *T. gondii* tissue cysts and the acquisition of the protozoon [52,53,54,55,56].

Regarding the cat’s environment, cats living in rural [45,53,57,58,59] or peri-urban/suburban areas [60,61] usually are subject to a significantly higher risk of infection. This is because rural and peri-urban cats come in contact with the *T. gondii* natural reservoirs that they hunt, such as infected prey (i.e., rodents), more frequently [45,57,60,61,62]. In addition, rural regions tend to have a higher oocyst burden in the environment (soil and water) compared to urban regions [58]. Our results from the chi-square tests are in concordance with these studies, as it was found that cats living in rural areas (villages) of Greece had significantly higher seropositivity than cats living in urban areas (cities).

According to our findings from chi-square tests and the binary logistic regression model, gender was not a significant risk factor for *T. gondii* seropositivity, as showcased in almost all previous studies [44,50,54,56,57,58,59,61,62,63,64,65,66,67,68,69,70,71]. Both male and female cats seem similarly vulnerable to acquiring *T. gondii*. A few studies which identified gender as a risk factor suggested that stray male cats have higher seropositivity than females. This finding might be explained because males consume more meals, and thus have a higher probability of becoming infected, and because they hunt in a larger area [72,73]. In particular, this difference in seropositivity was quite evident in regions with more prey intermediate hosts [72]. Conversely, some studies observed higher seropositivity in female cats [45,74,75,76]. The explanations provided were that females have different behaviours than males, and may wander more based on the season [45] or hormonal imbalances [76].

With regards to age, the risk of infection typically increases with cats’ age due to increased exposure, and not because older cats are more susceptible [59,77,78]. In this context, several studies recorded a significant increase in seropositivity as cats became older [44,45,52,53,54,56,57,58,59,65,66,67,69,70,72,73,74,78,79,80]. The only exception seems to be with kittens a few weeks old, as they may have a seroprevalence of 30% [66] or even 100% [76], probably due to the transfer of IgG maternal antibodies through the colostrum [64,76]. After that age, seropositivity starts to decrease before naturally increasing again after kittens become two months old and start hunting [66]. In our case, age was not a statistically significant risk factor both in the chi-square tests and in the binary logistic regression model, but the relative risk of seropositivity increased by 41% for cats older than six months compared to cats younger than six months. In detail, adult cats presented a seropositivity of 22.6% and young cats presented 16%, but the risk of infection was not significantly higher in adult cats, probably due to the small sample size of the young cats (*n* = 137). Moreover, the difference might not have been statistically significant due to the transfer of maternal antibodies in cats younger than six months, which increased their seropositivity. Similarly to our results, many researchers have not established any correlation between increased age and seroprevalence [50,61,63,64,71,76], although, in some of those studies, a non-significant increase in seropositivity in older cats was depicted [63,71].

Raw feeding has also been considered a significant risk factor for feline toxoplasmosis [52,54,57,74,78,81,82,83,84]. Cats consuming raw or undercooked viscera/meat may have a higher chance of ingesting tissue cysts containing bradyzoites [78]. Conversely, cats fed commercial diets have a lower risk of acquiring the infection. It has been shown that even cats that live indoors can have a high prevalence of *T. gondii* if they are fed a raw diet [65]. However, many researchers claim that raw meat diets are not always associated with a higher *T. gondii* seroprevalence in cats [50,53,56,59,69]. Our study’s results from the chi-square tests and the binary logistic regression model align with the latter findings since there was no statistically significant increase in seropositivity in raw-feeding cats. One possible explanation is that most owners stored the raw meat/viscera in the freezer for more than two days, allowing tissue cysts to be killed [85], a common practice for preserving meat in our country. Furthermore, the meat itself might not have been initially infected with *T. gondii* cysts, i.e., if it was beef which is generally considered safe [16].

Many previous studies consider outdoor access a key risk factor [52,53,57,59,70,80]. Our findings from the chi-square tests are consistent with the aforementioned studies; the risk of infection was significantly higher in cats with outdoor access, and the relative risk of infection was increased by 42%. Cats that freely roam outdoors hunt to eat, and their prey (rodents, birds, or even placentas/foetuses) might be infected or mechanically carrying oocysts (i.e., arthropods), compared with cats that live indoors and typically eat safe processed food [51,56,63]. In addition, cats that dwell outside have an increased chance of becoming infected with oocysts from the environment [53], i.e., when drinking contaminated water [27] or contacting contaminated trash [63]. Although some studies found no significant association between outdoor access and *T. gondii* seropositivity [58,69,74], almost all of them observed a slight increase in seropositivity in cats with outside access [69,74].

Finally, feral-stray cats usually have a higher risk of acquiring *T. gondii* than domiciled cats [52,65,80,86,87]. Stray cats play a major role in disseminating *T. gondii* oocysts in the environment and thus are of prime interest for the disease’s epidemiology. Considering their ability to overpopulate quickly, they pose an even greater risk [68]. However, in the present study, the risk of infection was not significantly different in stray cats than in client-owned cats, despite the higher seropositivity rate in stray cats. A possible explanation is that most client-owned cats also had outdoor access, such as those in a similar study [54], and were hunting. Alternatively, stray cats may only hunt occasionally and not feed directly from their prey, but rather from garbage containing human-prepared food that is cooked [27]. Furthermore, many owned cats in Greece derive from adoptions of shelter or stray cats. As a result, the risk of infection is not significantly different between client-owned and stray cats. Correspondingly, a few studies found no significant difference in the risk of infection between stray and client-owned cats [54,62,67,70,88].

## 5. Conclusions

*T. gondii* infections in cats are present in all nine geographic regions of Greece. In this large-scale study, the detection of anti-*T. gondii* IgG in cats was 21.8%. This finding implies that although a large portion of cats in Greece is seronegative, which translates to a relatively low environmental oocyst burden, *T. gondii* circulates among the feline population in the country. Therefore, our study highlights the importance of raising awareness to avoid infections. Towards this end, based on risk factor analyses, owners should limit their cats’ access outdoors and control their hunting habits, particularly in rural regions. The current research emphasizes the importance of updated seroprevalence estimation and risk factors studies which remain crucial within the One Health framework.

## Figures and Tables

**Table 1:** *Toxoplasma gondii* IgG seroprevalence in cats belonging to different risk factor groups.

Risk Factor	Risk Factor Group	Number of Tested Cats	Seroprevalence (%)	95% CI of Seroprevalence (%)
Environment	Living in rural areas (villages)	1090	23.3	20.8–25.8
	Living in urban areas (cities)	464	18.3	14.8–21.8
Gender	Female cats	799	23.5	20.6–26.4
	Male cats	755	20.0	17.2–22.9
Age	Adult cats (>6 months)	1329	22.6	20.4–24.9
	Young cats (≤6 months)	137	16.0	9.9–22.1
Raw feeding	Yes	745	22.6	19.6–25.6
	No	809	21.0	18.2–23.8
Lifestyle	Cats with outdoor access	1101	23.8	21.3–26.3
	Strictly indoor living cats	453	16.7	13.3–20.1
Ownership	Owned cats	805	20.6	17.8–23.4
	Stray cats	748	23.1	20.1–26.1
Hunting activity	Yes	992	24.3	21.6–27.0
	No	562	17.2	14.1–20.3
Total seroprevalence	-	1554	21.8	19.8–23.9

**Table 2:** Chi-square tests, odds ratios, and relatives risks for seropositivity of *T. gondii* in cats.

Variable	Group	% Seronegative (78.2%, *n* = 1215)	% Seropositive (21.8%, *n* = 339)	*χ* ^2^	df	*p*-Value	ϕ*_c_*	OR (95% CI)	RR
Environment	-	-	-	4.74	1	0.029	0.055	1.36 (1.03–1.78)	1.27
	Rural	68.8 (836)	74.9 (254)						
	Urban *	31.2 (379)	25.1 (85)						
Gender	-	-	-	2.84	1	0.092	0.043	1.23 (0.97–1.57)	1.18
	Female	50.3 (611)	55.5 (188)						
	Male *	49.7 (604)	44.5 (151)						
Age	-	-	-	3.14	1	0.076	0.046	1.53 (0.95–2.46)	1.41
	>6 months	89.9 (1028)	93.2 (301)						
	≤6 months *	10.1 (115)	6.8 (22)						
Raw feeding	-	-	-	0.64	1	0.426	0.020	1.10 (0.87–1.40)	1.08
	Yes	47.4 (576)	49.9 (169)						
	No *	52.6 (639)	50.1 (170)						
Lifestyle	-	-	-	9.51	1	0.002	0.078	1.56 (1.17–2.07)	1.42
	Outside	69.0 (838)	77.6 (263)						
	Inside *	31.0 (377)	22.4 (76)						
Ownership	-	-	-	1.43	1	0.232	0.030	1.16 (0.91–1.47)	1.12
	Owned	52.6 (639)	49.0 (166)						
	Stray *	47.4 (575)	51.0 (173)						
Hunting activity	-	-	-	10.71	1	0.001	0.083	1.55 (1.19–2.01)	1.41
	Yes	61.7 (750)	71.4 (242)						
	No *	38.3 (465)	28.6 (97)						

* Reference groups, df: degrees of freedom, ϕ*_c_*: Cramér’s V, OR: odds ratio, RR: relative risk.

**Table 3:** The association between the studied risk factors forced into the model (sex, age, raw feeding, and hunting activity) and the seropositivity to *Toxoplasma gondii*.

**Urban**	**B ^1^**	**S.E. ^2^**	**Wald**	***p***	**Odds Ratio**	**95% C.I. ^3^** **for EXP(B)**	
						**Lower**	**Upper**
<6 months	−0.59	0.407	2.09	0.148	0.56	0.25	1.23
≥6 months	*Ref.* ^4^						
Male	0.14	0.249	0.30	0.584	1.15	0.70	1.87
Female	*Ref.*						
No raw feeding	0.16	0.445	0.131	0.717	1.18	0.49	2.81
Raw feeding	*Ref.*						
No hunting activity	−0.82	0.313	6.84	0.009	0.44	0.24	0.81
Hunting activity	*Ref.*						
Constant	−0.97	0.400	5.84	0.016	0.38		
**Rural**	**B**	**S.E.**	**Wald**	***p***	**Odds ratio**	**95% C.I.** **for EXP(B)**	
						**Lower**	**Upper**
≤6 months	−0.25	0.313	0.64	0.424	0.78	0.42	1.44
>6 months	*Ref.*						
Male	−0.28	0.149	3.52	0.061	0.76	0.57	1.01
Female	*Ref.*						
No raw feeding	0.24	0.186	1.63	0.201	1.27	0.88	1.83
Raw feeding	*Ref.*						
No hunting activity	−0.26	0.251	1.11	0.293	0.77	0.47	1.26
Hunting activity	*Ref.*						
Constant	−1.09	0.111	95.95	0.000	0.34		

^1^ Beta coefficient, ^2^ standard error, ^3^ confidence interval, ^4^ reference groups.

**Table 4:** *Toxoplasma gondii* IgG seropositivity percentages in cats from different geographical regions of Greece.

Geographical Region	Number of Tested Cats	Seroprevalence (%)	95% CI of Seroprevalence (%)
Thrace	200	21.8	16.1–27.5
Macedonia	243	14.9	10.4–19.4
Epirus	181	18.7	13.0–24.4
Thessaly	171	18.5	12.7–24.3
Central Greece	179	21.0	15.0–27.0
Peloponnese	105	42.8	33.3–52.3
Crete	152	22.8	16.1–29.5
Ionian islands	170	20.0	14.0–26.0
Aegean islands	153	26.6	19.6–33.6
**Total:**	**1554**	**21.8**	**19.8–23.9**

## Data Availability

Not applicable.

## References

[1] Mehlhorn H. (2016). Encyclopedia of Parasitology.

[2] Dubey J.P. (2008). The history of *Toxoplasma gondii*—The first 100 years. J. Eukaryot. Microbiol..

[3] Tenter A.M., Heckeroth A.R., Weiss L.M. (2000). *Toxoplasma gondii*: From animals to humans. Int. J. Parasitol..

[4] Dubey J.P., Lindsay D.S., Speer C.A. (1998). Structures of *Toxoplasma gondii* tachyzoites, bradyzoites, and sporozoites and biology and development of tissue cysts. Clin. Microbiol. Rev..

[5] Almeria S., Dubey J.P. (2021). Foodborne transmission of *Toxoplasma gondii* infection in the last decade. An overview. Res. Vet. Sci..

[6] Atmaca H.T., Dincel G.C., Macun H.C., Terzi O.S., Uzunalioglu T., Kalender H., Kul O. (2013). A rare case of feline congenital *Toxoplasma gondii* infection: Fatal outcome of systemic toxoplasmosis for the mother and its kitten. Berl. Munch. Tierarztl. Wochenschr..

[7] Davidson M.G. (2000). Toxoplasmosis. Vet. Clin. N. Am.—Small Anim. Pract..

[8] Calero-Berna R., Gennari S.M. (2019). Clinical toxoplasmosis in dogs and cats: An update. Front. Vet. Sci..

[9] Schlüter D., Barragan A. (2019). Advances and challenges in understanding cerebral toxoplasmosis. Front. Immunol..

[10] Wang Z.D., Wang S.C., Liu H.H., Ma H.Y., Li Z.Y., Wei F., Zhu X.Q., Liu Q. (2017). Prevalence and burden of *Toxoplasma gondii* infection in HIV-infected people: A systematic review and meta-analysis. Lancet HIV.

[11] Machumi I., M Mirambo M., Ruganuza D., Rambau P., Massinde A.N., Kihunrwa A., Mshana S.E., Morona D. (2017). Factors Associated With *Toxoplasma gondii* IgG and IgM Antibodies, and Placental Histopathological Changes Among Women With Spontaneous Abortion in Mwanza City, Tanzania. East Afr. Health Res. J..

[12] de Moraes É.P.B.X., da Costa M.M., Dantas A.F.M., da Silva J.C.R., Mota R.A. (2011). *Toxoplasma gondii* diagnosis in ovine aborted fetuses and stillborns in the State of Pernambuco, Brazil. Vet. Parasitol..

[13] Sakamoto C.A.M., Da Costa A.J., Gennari S.M., Pena H.F.J., Toniollo G.H., Lopes W.D.Z., Bichuette M.A., Betini C.M., Amarante A.F.T., Bresciani K.D.S. (2009). Experimental infection of pregnant queens with two major Brazilian clonal lineages of *Toxoplasma gondii*. Parasitol. Res..

[14] Montoya J.G., Liesenfeld O. (2004). Toxoplasmosis. Lancet.

[15] World Health Organization. https://www.euro.who.int/__data/assets/pdf_file/0005/402989/50607-WHO-Food-Safety-publicationV4_Web.pdf.

[16] Beugnet F., Halos L., Guillot J. (2018). Textbook of Clinical Parasitology in Dogs and Cats.

[17] Davidson M.G., Lappin M.R., Rottman J.R., Tompkins M.B., English R.V., Bruce A.T., Jayawickrama J. (1996). Paradoxical effect of clindamycin in experimental, acute toxoplasmosis in cats. Antimicrob. Agents Chemother..

[18] Montoya J.G. (2002). Laboratory diagnosis of *Toxoplasma gondii* infection and toxoplasmosis. J. Infect. Dis..

[19] Dubey J.P., Cerqueira-Cézar C.K., Murata F.H.A., Kwok O.C.H., Yang Y.R., Su C. (2020). All about toxoplasmosis in cats: The last decade. Vet. Parasitol..

[20] Lappin M.R. (1996). Feline toxoplasmosis: Interpretation of diagnostic test results. Semin. Vet. Med. Surg.-Small Anim..

[21] Huang X., Xuan X., Hirata H., Yokoyama N., Xu L., Suzuki N., Igarashi I. (2004). Rapid Immunochromatographic Test Using Recombinant SAG2 for Detection of Antibodies against *Toxoplasma gondii* in Cats. J. Clin. Microbiol..

[22] Jiang W., Liu Y., Chen Y., Yang Q., Chun P., Yao K., Han X., Wang S., Yu S., Liu Y. (2015). A novel dynamic flow immunochromatographic test (DFICT) using gold nanoparticles for the serological detection of *Toxoplasma gondii* infection in dogs and cats. Biosens. Bioelectron..

[23] Ybañez R.H.D., Kyan H., Nishikawa Y. (2020). Detection of antibodies against *Toxoplasma gondii* in cats using an immunochromatographic test based on GRA7 antigen. J. Vet. Med. Sci..

[24] Dubey J.P., Frenkel J.K. (1972). Cyst-Induced Toxoplasmosis in Cats. J. Protozool..

[25] Dumètre A., Dardé M.L. (2003). How to detect *Toxoplasma gondii* oocysts in environmental samples?. FEMS Microbiol. Rev..

[26] Yan C., Fu L.L., Yue C.L., Tang R.X., Liu Y.S., Lv L., Shi N., Zeng P., Zhang P., Wang D.H. (2012). Stray dogs as indicators of *Toxoplasma gondii* distributed in the environment: The first report across an urban-rural gradient in China. Parasites Vectors.

[27] Meireles L.R., Galisteo A.J., Pompeu E., Andrade H.F. (2004). *Toxoplasma gondii* spreading in an urban area evaluated by seroprevalence in free-living cats and dogs. Trop. Med. Int. Health.

[28] Symeonidou I., Gelasakis A.I., Arsenopoulos K., Angelou A., Beugnet F., Papadopoulos E. (2018). Feline gastrointestinal parasitism in Greece: Emergent zoonotic species and associated risk factors. Parasites Vectors.

[29] Montazeri M., Mikaeili Galeh T., Moosazadeh M., Sarvi S., Dodangeh S., Javidnia J., Sharif M., Daryani A. (2020). The global serological prevalence of *Toxoplasma gondii* in felids during the last five decades (1967-2017): A systematic review and meta-analysis. Parasites Vectors.

[30] Bellows J., Center S., Daristotle L., Estrada A.H., Flickinger E.A., Horwitz D.F., Lascelles B.D.X., Lepine A., Perea S., Scherk M. (2016). Aging in cats: Common physical and functional changes. J. Feline Med. Surg..

[31] Shin S. (2022). Personal communication.

[32] Kostopoulou D., Claerebout E., Arvanitis D., Ligda P., Voutzourakis N., Casaert S., Sotiraki S. (2017). Abundance, zoonotic potential and risk factors of intestinal parasitism amongst dog and cat populations: The scenario of Crete, Greece. Parasites Vectors.

[33] Dubey J.P. (2021). Toxoplasmosis of Animals and Humans.

[34] Zhu S., Shapiro K., VanWormer E. (2021). Dynamics and epidemiology of *Toxoplasma gondii* oocyst shedding in domestic and wild felids. Transbound. Emerg. Dis..

[35] Schares G., Vrhovec M.G., Pantchev N., Herrmann D.C., Conraths F.J. (2008). Occurrence of *Toxoplasma gondii* and *Hammondia hammondi* oocysts in the faeces of cats from Germany and other European countries. Vet. Parasitol..

[36] Verma S.K., Cerqueira-Cézar C.K., Murata F.H.A., Lovallo M.J., Rosenthal B.M., Dubey J.P. (2017). Bobcats (*Lynx rufus*) are natural definitive host of *Besnoitia darlingi*. Vet. Parasitol..

[37] Hatam-Nahavandi K., Calero-Bernal R., Rahimi M.T., Pagheh A.S., Zarean M., Dezhkam A., Ahmadpour E. (2021). *Toxoplasma gondii* infection in domestic and wild felids as public health concerns: A systematic review and meta-analysis. Sci. Rep..

[38] Montoya A., Miró G., Mateo M., Ramírez C., Fuentes I. (2008). Molecular characterization of *Toxoplasma gondii* isolates from cats in Spain. J. Parasitol..

[39] Hotea I., Oprescu I., Ilie M., Imre K., Imre M., Darabus G. (2011). Seroprevalence of *Toxoplasma gondii* infection in cats and sheep in Arad County. Med. Vet..

[40] Lamaj S., Dhamo G., Dova I. (2015). *Toxoplasma gondii* Infection in cats from southwest areas of Albania. Albanian J. Agric. Sci..

[41] Can H., Döşkaya M., Ajzenberg D., Özdemir H.G., Caner A., Iz S.G., Döşkaya A.D., Atalay E., Çetinkaya Ç., Ürgen S. (2014). Genetic characterization of *Toxoplasma gondii* isolates and toxoplasmosis seroprevalence in stray cats of Izmir, Turkey. PLoS ONE.

[42] de Jesus Pena H.F., Evangelista C.M., Casagrande R.A., Biezus G., Wisser C.S., Ferian P.E., de Moura A.B., Rolim V.M., Driemeier D., Oliveira S. (2017). Fatal toxoplasmosis in an immunosuppressed domestic cat from Brazil caused by *Toxoplasma gondii* clonal type I. Rev. Bras. Parasitol. Vet..

[43] Dubey J.P., Lappin M.R., Kwok O.C.H., Mofya S., Chikweto A., Baffa A., Doherty D., Shakeri J., MacPherson C.N.L., Sharma R.N. (2009). Seroprevalence of *Toxoplasma gondii* and concurrent *Bartonella* spp., Feline Immunodeficiency Virus, and Feline Leukemia Virus infections in cats from Grenada, West Indies. J. Parasitol..

[44] Pena H.F.J., Soares R.M., Amaku M., Dubey J.P., Gennari S.M. (2006). *Toxoplasma gondii* infection in cats from São Paulo state, Brazil: Seroprevalence, oocyst shedding, isolation in mice, and biologic and molecular characterization. Res. Vet. Sci..

[45] Hornok S., Edelhofer R., Joachim A., Farkas R., Berta K., Répási A., Lakatos B. (2008). Seroprevalence of *Toxoplasma gondii* and *Neospora caninum* infection of cats in Hungary. Acta Vet. Hung..

[46] Hartmann K., Addie D., Belák S., Boucraut-Baralon C., Egberink H., Frymus T., Gruffydd-Jones T., Hosie M.J., Lloret A., Lutz H. (2013). *Toxoplasma Gondii* Infection in cats: ABCD guidelines on prevention and management. J. Feline Med. Surg..

[47] Dubey J.P., Lappin M.R., Thulliez P. (1995). Long-term antibody responses of cats fed *Toxoplasma gondii* tissue cysts. J. Parasitol..

[48] Malmasi A., Mosallanejad B., Mohebali M., Sharifian Fard M., Taheri M. (2009). Prevention of shedding and re-shedding of *Toxoplasma gondii* oocysts in experimentally infected cats treated with oral clindamycin: A preliminary study. Zoonoses Public Health.

[49] Samad A., Islam M.R., Dey B.C., Alam M.M. (1997). Effects of corticosteroids in stray cats with natural antibodies to *Toxoplasma gondii*. J. Protozool. Res..

[50] Castillo-Morales V.J., Acosta Viana K.Y., Guzmán-Marín E.D.S., Jiménez-Coello M., Segura-Correa J.C., Aguilar-Caballero A.J., Ortega-Pacheco A. (2012). Prevalence and risk factors of *Toxoplasma gondii* infection in domestic cats from the tropics of Mexico using serological and molecular tests. Interdiscip. Perspect. Infect. Dis..

[51] Jones J.L., Dubey J.P. (2010). Waterborne toxoplasmosis—Recent developments. Exp. Parasitol..

[52] Opsteegh M., Haveman R., Swart A.N., Mensink-Beerepoot M.E., Hofhuis A., Langelaar M.F.M., van der Giessen J.W.B. (2012). Seroprevalence and risk factors for *Toxoplasma gondii* infection in domestic cats in The Netherlands. Prev. Vet. Med..

[53] Must K., Lassen B., Jokelainen P. (2015). Seroprevalence of and risk factors for *Toxoplasma gondii* infection in cats in Estonia. Vector-Borne Zoonotic Dis..

[54] Feitosa T.F., Vilela V.L.R., Dantas E.S., Souto D.V.O., Pena H.F.J., Athayde A.C.R., Azevêdo S.S. (2014). *Toxoplasma gondii* and *Neospora caninum* in domestic cats from the Brazilian semi-arid: Seroprevalence and risk factors. Arq. Bras. Med. Vet. Zootec..

[55] Kulasena V.A., Rajapakse R.P.V.J., Dubey J.P., Dayawansa P.N., Premawansa S. (2011). Seroprevalence of *Toxoplasma gondii* in cats from Colombo, Sri Lanka. J. Parasitol..

[56] Fernández F., Ouviña G., Clot E., Fernandes Guido R., Codoni C. (1995). Prevalence of *Toxoplasma gondii* antibodies in cats in the western part of Great Buenos Aires, Argentina, 1993. Vet. Parasitol..

[57] Györke A., Opsteegh M., Mircean V., Iovu A., Cozma V. (2011). *Toxoplasma gondii* in Romanian household cats: Evaluation of serological tests, epidemiology and risk factors. Prev. Vet. Med..

[58] Ahmad N., Ahmed H., Irum S., Qayyum M. (2014). Seroprevalence of IgG and IgM antibodies and associated risk factors for toxoplasmosis in cats and dogs from sub-tropical arid parts of Pakistan. Trop. Biomed..

[59] Rosa L.D., de Moura A.B., Trevisani N., Medeiros A.P., Sartor A.A., da Souza A.P., Bellato V. (2010). *Toxoplasma gondii* antibodies on domiciled cats from Lages municipality, Santa Catarina State, Brazil. Rev. Bras. Parasitol. Vet..

[60] Munhoz A.D., Hage S.B., Cruz R.D.S., Calazans A.P.F., Silva F.L., Albuquerque G.R., Lacerda L.C. (2017). Toxoplasmosis in cats in northeastern Brazil: Frequency, associated factors and coinfection with *Neospora caninum*, Feline Immunodeficiency Virus and Feline Leukemia Virus. Vet. Parasitol. Reg. Stud. Rep..

[61] Oi M., Yoshikawa S., Maruyama S., Nogami S. (2015). Comparison of *Toxoplasma gondii* seroprevalence in shelter cats and dogs during 1999–2001 and 2009–2011 in Tokyo, Japan. PLoS ONE.

[62] DeFeo M.L., Dubey J.P., Mather T.N., Rhodes R.C. (2002). Epidemiologic investigation of seroprevalence of antibodies to *Toxoplasma gondii* in cats and rodents. Am. J. Vet. Res..

[63] Ayinmode A.B., Oluwayelu D.O., Babalola E.T., Lawani M.A. (2017). Serologic survey of *Toxoplasma gondii* antibodies in cats (*Felis catus*) sold at live animal markets in southwestern Nigeria. Bulg. J. Vet. Med..

[64] Gauss C.B.L., Almería S., Ortuño A., Garcia F., Dubey J.P. (2003). Seroprevalence of *Toxoplasma gondii* Antibodies in Domestic Cats from Barcelona, Spain. J. Parasitol..

[65] Salant H., Spira D.T. (2004). A cross-sectional survey of anti-*Toxoplasma gondii* antibodies in Jerusalem cats. Vet. Parasitol..

[66] Jakob-Hoff R.M., Dunsmore J.D. (1983). Epidemiological aspects of toxoplasmosis in southern Western Australia. Aust. Vet. J..

[67] Lee S.E., Kim N.H., Chae H.S., Cho S.H., Nam H.W., Lee W.J., Kim S.H., Lee J.H. (2011). Prevalence of *Toxoplasma gondii* Infection in Feral Cats in Seoul, Korea. J. Parasitol..

[68] Lopes A.P., Oliveira A.C., Granada S., Rodrigues F.T., Papadopoulos E., Schallig H., Dubey J.P., Cardoso L. (2017). Antibodies to *Toxoplasma gondii* and *Leishmania* spp. in domestic cats from Luanda, Angola. Vet. Parasitol..

[69] Deksne G., Petruseviča A., Kirjušina M. (2013). Seroprevalence and factors associated with *Toxoplasma gondii* infection in domestic cats from urban areas in Latvia. J. Parasitol..

[70] da Silva Rocha Fournier G.F., Lopes M.G., Marcili A., Ramirez D.G., Acosta I.C.L., da Silva Ferreira J.I.G., Cabral A.D., de Lima J.T.R., de Jesus Pena H.F., Dias R.A. (2014). Toxoplasma gondii in domestic and wild animals from forest fragments of the municipality of Natal, northeastern Brazil. Rev. Bras. Parasitol. Vet..

[71] Arene F.O.I. (1984). The prevalence and public health significance of *Toxoplasma gondii* in domestic cats in the Niger Delta. Public Health.

[72] Afonso E., Thulliez P., Pontier D., Gilot-Fromont E. (2007). Toxoplasmosis in prey species and consequences for prevalence in feral cats: Not all prey species are equal. Parasitology.

[73] Vollaire M.R., Radecki S.V., Lappin M.R. (2005). Seroprevalence of *Toxoplasma gondii* antibodies in clinically ill cats in the United States. Am. J. Vet. Res..

[74] Besné-Mérida A., Figueroa-Castillo J.A., Martínez-Maya J.J., Luna-Pastén H., Calderón-Segura E., Correa D. (2008). Prevalence of antibodies against *Toxoplasma gondii* in domestic cats from Mexico City. Vet. Parasitol..

[75] Jittapalapong S., Nimsupan B., Pinyopanuwat N., Chimnoi W., Kabeya H., Maruyama S. (2007). Seroprevalence of *Toxoplasma gondii* antibodies in stray cats and dogs in the Bangkok metropolitan area, Thailand. Vet. Parasitol..

[76] Dhamraa R., Alwan J. (2014). Seropathological Diagnosis of *Toxoplasma gondii* in Stray Cats in Baghdad Province. Iraqi J. Vet. Med..

[77] Dubey J.P., Lindsay D.S., Lappin M.R. (2009). Toxoplasmosis and other intestinal coccidial infections in cats and dogs. Vet. Clin. North Am.—Small Anim. Pract..

[78] Must K., Hytönen M.K., Orro T., Lohi H., Jokelainen P. (2017). *Toxoplasma gondii* seroprevalence varies by cat breed. PLoS ONE.

[79] Childs J.E., Rooney J.A., Cooper J.L., Olson J.G., Regnery R.L. (1994). Epidemiologic observations on infection with *Rochalimaea* species among cats living in Baltimore, Md. J. Am. Vet. Med. Assoc..

[80] Nutter F.B., Dubey J.P., Levine J.F., Breitschwerdt E.B., Ford R.B., Stoskopf M.K. (2004). Seroprevalences of antibodies against *Bartonella henselae* and *Toxoplasma gondii* and fecal shedding of *Cryptosporidium* spp., *Giardia* spp., and *Toxocara cati* in feral and pet domestic cats. J. Am. Vet. Med. Assoc..

[81] Lucas S.R.R., Hagiwara M.K., De Loureiro V.S., Ikesaki J.Y.H., Birgel E.H. (1999). *Toxoplasma gondii* infection in Brazilian domestic outpatient cats. Rev. Inst. Med. Trop. Sao Paulo.

[82] Smielewska-Loś E., Pacoń J. (2002). *Toxoplasma gondii* infection of cats in epizootiological and clinical aspects. Pol. J. Vet. Sci..

[83] Lopes A.P., Cardoso L., Rodrigues M. (2008). Serological survey of *Toxoplasma gondii* infection in domestic cats from northeastern Portugal. Vet. Parasitol..

[84] Jokelainen P., Simola O., Rantanen E., Näreaho A., Lohi H., Sukura A. (2012). Feline toxoplasmosis in Finland: Cross-sectional epidemiological study and case series study. J. Vet. Diagn. Investig..

[85] El-Nawawi F.A., Tawfik M.A., Shaapan R.M. (2008). Methods for inactivation of *Toxoplasma gondii* cysts in meat and tissues of experimentally infected sheep. Foodborne Pathog. Dis..

[86] Knaus B.U., Fehler K. (1989). *Toxoplasma gondii* infections and oocyst shedding in domestic cats and the significance of this for the epidemiology and epizootiology of toxoplasmosis. Angew. Parasitol..

[87] Al-Mohammed H.I. (2011). Seroprevalence of *Toxoplasma* gondii infection in cats, dogs and ruminant animals in Al-Ahsa area in Saudi Arabia. Res. J. Med. Sci..

[88] Advincula C., Yaser Perez Iewida S., Salibay C.C., Karla J., Cabanacan-Salibay C. (2010). Serologic detection of *Toxoplasma gondii* infection in stray and household cats and its hematologic evaluation. Sci. Med. (Porto Alegre).

